# Dietary Curcumin Improves Energy Metabolism, Brain Monoamines, Carcass Traits, Muscle Oxidative Stability and Fatty Acid Profile in Heat-Stressed Broiler Chickens

**DOI:** 10.3390/antiox10081265

**Published:** 2021-08-09

**Authors:** Ayman S. Salah, Omar A. Ahmed-Farid, Mohamed Abdo Nassan, Mahmoud S. El-Tarabany

**Affiliations:** 1 Department of Animal Nutrition and Clinical Nutrition, Faculty of Veterinary Medicine, New Valley University, El-Kharga P.O. Box 72511, Egypt; asabry3999@yahoo.com; 2 Physiology Department, National Organization for Drug Control and Research (NODCAR), Giza P.O. Box 35521, Egypt; ebntaimya@yahoo.com; 3 Department of Clinical Laboratory Sciences, Turabah University College, Taif University, P.O. Box 11099, Taif 21944, Saudi Arabia; m.nassan@tu.edu.sa; 4 Department of Animal Wealth Development, Faculty of Veterinary Medicine, Zagazig University, Sharkia P.O. Box 44511, Egypt

**Keywords:** broilers, heat stress, metabolism, energy, nuerotransmittors

## Abstract

The aim of the present study was to elucidate the impacts of dietary curcumin supplementation on energy metabolism, brain monoamines and muscle oxidative stability in heat-stressed broilers. In total, 120 day-old chicks were allocated into three equal groups of four replicates. The first group (T_1_) was maintained on a thermoneutral condition, while the second group (T_2_) was subjected to 8 h of thermal stress (34 °C), and both groups fed the basal diet with no supplement. The third group (T_3_) was exposed to the same thermal stress conditions and fed the basal diet supplemented with curcumin (100 mg kg^−1^ diet). The dietary curcumin supplementation significantly increased the breast yield (*p* = 0.004), but reduced the percentage of abdominal fat (*p* = 0.017) compared with the T_2_ group. The addition of curcumin to broiler diets significantly improved the levels of monounsaturated fatty acids (MUFAs) and polyunsaturated fatty acids (PUFAs) in breast and thigh muscles compared with the T_2_ group (*p* < 0.05). The curcumin-supplemented group showed significantly lower levels of malondialdehyde in the breast and thigh muscles than that of the T_2_ group (*p* = 0.001 and 0.015, respectively). The dietary curcumin supplementation significantly improved the levels of ATP and CoQ10 in liver tissues (*p* = 0.012 and 0.001, respectively) and brain serotonin (*p* = 0.006) as compared to the T2 group. Meanwhile, the heat-stressed group showed significantly higher levels of ADP and Na,K-ATPase in the liver tissues than that of the other experimental groups (*p* = 0.011 and 0.027, respectively). It could be concluded that dietary curcumin supplementation may improve carcass yield, energy biomarkers, brain serotonin and muscle oxidative stability of heat-stressed broiler chickens.

## 1. Introduction

In the tropics and subtropics, heat stress has been considered as the major limiting factor of poultry industry [1]. Moreover, the expected increase in global warming will exaggerate heat stress-related hazards [2]. Indeed, it has been reported that exposure to thermal stress adversely affects survival [3], feed intake [4], growth performance [5], welfare [6], profitability [7] and meat quality [8] of broiler chickens. The higher susceptibility of broiler chickens to thermal stress is usually attributed to their rapid growth rate and high metabolic heat production [9].

At the cellular level, heat stress adversely affects the functional structures of membranes and cellular organelles [10]. Furthermore, thermal stress activates lipid peroxidation process in blood and tissues [11]. In this context, heat stress usually increases the production of reactive oxygen species (ROS) in mitochondria, and consequently disturbs the energy metabolism and ATP synthesis [12]. Mujahid et al. [13] suggested that mitochondria are more susceptible to oxidative damage, probably due to the increased contents of polyunsaturated fatty acids in their membranes. They also indicated that acute thermal stress increases the oxidative damages in the skeletal muscles of broilers. In addition, heat stress accelerates the denaturation process of proteins and oxidation of lipids within the muscles, deteriorating the quality parameters and nutritive value of broiler meat [14]. Lu et al. [15] also showed that prolonged heat stress alters the process of glycolysis and intramuscular fat deposition in broilers.

Several strategies have suggested modifications on housing design, cooling systems and diet composition to mitigate the harmful effects of thermal stress in poultry [16]. Recently, herbal plants have been used to ameliorate the negative effects of thermal stress in poultry. Curcumin is the main active ingredient in *Curcuma longa* (turmeric herbal plant) and is of high economic value due to its peculiar rhizome [17]. Furthermore, curcumin exhibited a protective effect against the lipid peroxidation process maintaining the stability of cellular organelles through free radicals’ scavenging [18]. In this context, several trials confirmed the pharmaceutical activities of curcumin including anti-inflammatory [19], antioxidant [20] and antimicrobial [21] properties. However, the literature related to the effect of curcumin supplementation on performance and physiological responses of heat-stressed broiler chickens is relatively limited. Therefore, the aim of the present study is to elucidate the impact of dietary curcumin supplementation on energy metabolism, brain monoamines, carcass traits, muscle oxidative stability and fatty acid profile of broiler chickens subjected to a prolonged thermal stress.

## 2. Materials and Methods

### 2.1. Birds and Management

In total, 120 day-old male chicks (Ross strain) were allocated into three equal groups of four replicates (10 birds/replicate). The chicks were housed in commercial pens (15 birds/m^2^) covered with fresh wood shavings. All birds had free access to feed and water. Regular supplementation of heat was performed by digital diesel heaters to maintain a stable housing temperature (Naganpuriya High Tech Farming Equipment). The house environment was maintained at 34 °C during the first week of age. Thereafter, and gradually, temperature was declined to reach 23 °C at the day 21 of age. The first group (T_1_) was maintained on a thermoneutral condition (23 ± 1.5 °C) and fed the basal diet with no supplements, while the second group (T_2_) was exposed to 8 h of continuous thermal stress at 34 °C (08:00–16:00) and 23 ± 1 °C for the remaining time, and also fed the basal diet with no supplements. The third group (T_3_) was exposed to the same thermal stress conditions and fed a basal diet supplemented with curcumin (100 mg kg^−1^ diet). The curcumin were purchased from Sigma-Aldrich (Sigma, St. Louis, MO, USA). This level of dietary curcumin supplementation was adjusted according to Rajput et al. [22], who stated that 100 mg kg^−1^ is the minimal supplemental level of curcumin for broiler diets. The relative humidity of experimental pens was adjusted at 58 ± 3%. A routine vaccination program against Newcastle disease and Gumboro disease was applied. According to the guidelines of NRC [23], all birds fed the same starter and grower-finisher diets (Table 1). On a weekly basis, body weight and feed intake were recorded to determine the average daily feed intake (ADFI).

### 2.2. Carcass Traits

At the end of the experiment (42 days), 12 birds were selected from each group (3 birds per each replicate), weighed and slaughtered to evaluate carcass traits. The slaughter technique followed the pre-stunning method and guidelines of the Malaysian institutes [24]. Carcasses were eviscerated, spray-washed and chilled for 30 min at 2 °C. The dressing percentage was estimated as an actual carcass weight relative to the final body weight. Thereafter, carcasses were divided into different cuts (breast and legs). The relative weight of breast, legs, liver, heart and abdominal fat were calculated.

### 2.3. Determination of Muscle Fatty Acid Profiles

From the above chilled carcasses, the thigh and breast muscles were carefully dissected and any fat or connective tissues were removed. The lean muscle samples were directed to the chemical analyses of muscle fatty acids (FAs) profiles. A chloroform:methanol mixture (2:1) was used to extract the lipids from muscle samples and the free FAs were purchased from Sigma-Aldrich (Sigma, St. Louis, MO, USA). The esterification process of supernatant was initiated by adding 2 mL of methanol:sulphuric acid mixture (95:5). The gas chromatography (Agilent Technologies-7890A GC) was used to finalize the analysis. The injection of samples into the GC set loop was performed using the SupelcoSP2330 column, (30 mm × 0.32 mm × 0.2 μm film thickness; Cat. No. 24073, Sigma-Aldrich, St. Louis, MO, USA). The conditions of flow rate through the GC column and the splitless injection mode were practiced according to the procedures described by Radwan and Ahmed [25]. The levels of muscle saturated fatty acids, monounsaturated fatty acids (MUFAs) and polyunsaturated fatty acids (PUFAs) are illustrated as g/100 g.

### 2.4. Determination of Muscle Malondialdehyde

The breast and thigh muscles were homogenized in buffer solution (100 mol/L KCl, 50 mol/L Tris–HCl, and 2 mol/L EGTA, pH 7.4), and centrifuged at 700× *g* to collect a clear supernatant layer in order to determine the malondialdehyde (MDA) concentrations. The HPLC Agilent technology (HP 1100 series, Santa Clara, CA, USA) was used to quantify the concentration of MDA in muscle samples [26]. The analytical column of Supelcosil C18 (5 µm particle and 80 Ao pore size) was used at a flow rate of 1.5 mL/min and 250 nm wavelength.

### 2.5. Determination of Brain Monoamines

Brain samples (striatum, frontal cortex, and hypothalamus) were homogenized in a HPLC-grade methanol solution [27]. After the derivatization process was completed, a diluent composed of 0.71-g disodium-hydrogen phosphate (pH of 7.4) plus 5% acetonitrile was mixed with the dried samples. For 10 min, the homogenate of each sample was centrifuged (1792× *g*) and the supernatant was collected. The level of brain serotonin was determined by the HPLC in accordance with the method described by Pagel et al. [28]. Compared with the standard, the resulting chromatogram had to characterize the concentration of serotonin (5-HT) and 5-hydroxyindoleacetic acid (5-HIAA) as μg per gram of brain tissue.

### 2.6. Determination of CoQ10 and Energy Biomarkers in Liver Tissues

Liver adenosine tri- and di-phosphate (ATP and ADP) were quantified by the HPLC [29]. Fifty hundred microliters of liver tissue was homogenized with ice cold 10% potassium chloride then centrifuged at 2800× *g* for 20 min to collect a clear supernatant. For deprotonization, 200 µL of supernatant was mixed with 1 mL of methanol (70%) and prepared for HPLC analysis (C-18 Spherclone column). The reports and chromatograms were obtained from chemstation program, with Agilent at wave length of 254 nm and injection volume of 20 µL. Additionally, the level of liver CoQ10 was quantified by the HPLC according to the modified protocol of Niklowitz et al. [30].

### 2.7. Statistical Analyses

The data were analyzed by ANOVA procedures of the IBM SPSS software program (Version 16.0; IBM Corp., Armonk, NY, USA). The model included the fixed effects of the thermal treatment (three levels: T_1_, T_2_ and T_3_) and the random effect of experimental error. Using the Duncan Multiple Range Test (DMRT), multiple mean comparisons were performed. The outputs are expressed as means and the standard error of means (SEM).

## 3. Results

The impact of dietary curcumin supplementation on feed intake and carcass traits of heat-stressed broiler chickens are presented in Table 2. The dietary curcumin supplementation significantly improved the ADFI of heat-stressed broiler chickens (*p* = 0.048). At the same time, the dietary curcumin supplementation significantly increased the dressing percentage and breast yield (*p* = 0.010 and 0.004, respectively), but reduced the percentage of abdominal fat (*p* = 0.017) as compared to the T_2_ group. Meanwhile, the percentages of leg, liver and heart did not differ among the experimental groups (*p* = 0.070, 0.099 and 0.269, respectively).

The impact of dietary curcumin supplementation on FA profiles of the breast and thigh muscles in heat-stressed broiler chickens are illustrated in Table 3; Table 4, respectively. The dietary curcumin supplementation significantly reduced the levels of saturated FAs in breast (myristic and palmitic) and thigh (palmitic and stearic) muscles as compared to the T_2_ group (*p* < 0.05). Meanwhile, the addition of curcumin to broiler diets significantly improved the levels of MUFAs (myristoleic, palmitoleic and Oleic) and PUFAs (linoleic, docosahexaenoic and eicosapentaenoic) in breast and thigh muscles as compared to the T_2_ group (*p* < 0.05). The levels of α- linolenic acid in the breast and thigh muscles did not differ among the experimental groups (*p* = 0.093, and 0.086, respectively).

**Figure 1:**
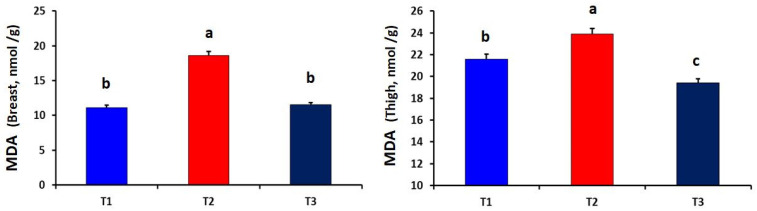
Effect of dietary curcumin supplementation on the levels of malondialdehyde (MDA) in the breast and thigh muscles of heat-stressed broiler chickens (*p* = 0.001 and 0.015, respectively). a,b,c Values within a row with different superscripts differ significantly.

As described in Figure 1, the curcumin-supplemented group showed significantly lower levels of MDA in the breast and thigh muscles as compared to the T_2_ group (*p* = 0.001 and 0.015, respectively).

**Figure 2:**
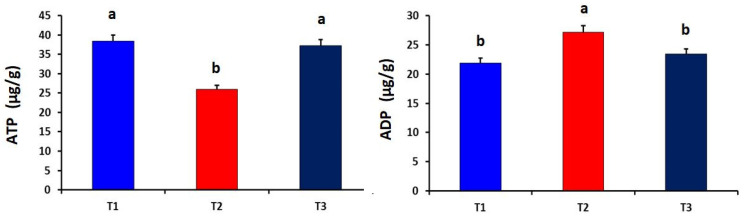
Effect of dietary curcumin supplementation on the levels of adenosine triphosphate (ATP) and adenosine diphosphate (ADP) in liver tissues of heat-stressed broiler chickens (*p* = 0.001 and 0.011, respectively). a,b Values within a row with different superscripts differ significantly.

The dietary curcumin supplementation significantly (*p* = 0.001) improved the levels of ATP in liver tissues as compared to the T_2_ group (Figure 2).

**Figure 3:**
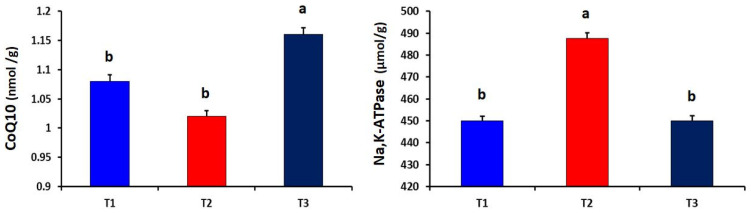
Effect of dietary curcumin supplementation on the levels of Coenzyme Q10 (CoQ10) and Na,K-ATPase in liver tissues of heat-stressed broiler chickens (*p* = 0.012 and 0.027, respectively). a,b Values within a row with different superscripts differ significantly.

**Figure 4:**
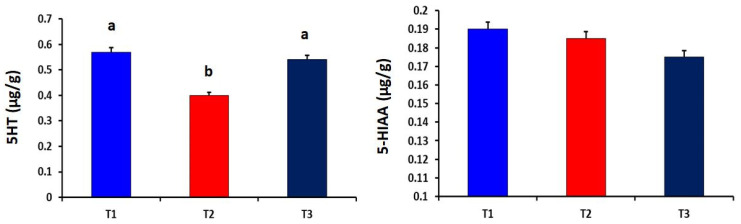
Effect of dietary curcumin supplementation on the levels of serotonin (5-HT) and 5-hydroxyindoleacetic acid (5-HIAA) in brain tissues of heat-stressed broiler chickens (*p* = 0.006 and 0.132, respectively). a,b Values within a row with different superscripts differ significantly.

Meanwhile, the heat-stressed (T_2_) group showed significantly higher levels of ADP and Na,K-ATPase in the liver tissues than that of the other experimental groups (*p* = 0.011 and 0.027, respectively). The dietary curcumin supplementation significantly (*p* = 0.012) increased the CoQ10 level in liver tissues as compared to other experimental groups (Figure 3). Compared to the heat-stressed group, the T_1_ and T_2_ groups showed significantly higher serotonin in brain tissues of broiler chickens (*p* = 0.006). However, the level of 5-hydroxyindoleacetic acid in brain tissues did not differ among the experimental groups (Figure 4).

## 4. Discussion

It is widely accepted that broiler chickens are more susceptible to adverse environmental conditions, perhaps due to their continuous selection for rapid growth rate [31]. However, the majority of literature has focused on acute form, either cyclic or continuous thermal stress. In the present study, dietary curcumin supplementation significantly improved the ADFI of heat-stressed broiler chickens. This finding is probably attributed to the ability of curcumin supplement to improve the intestinal barrier and mitochondrial functions [32]. Herein, curcumin also increased the level of serotonin in brain tissues of heat-stressed broilers, which maintain the gastrointestinal motility and visceral sensation [33]. Consistent with our findings, Gowda et al. [34] suggested that the supplementation of curcuma to broiler diet (0.5–1%) significantly improved the feed intake. Recently, others suggested that the addition of curcumin to growing diet at 400 mg/kg enhanced the feed intake of ochratoxin-exposed White Pekin ducklings [32]. On the contrary, Rajput et al. [22] noticed that the supplementation of curcumin to broiler diets did not influence the feed intake.

In the current study, dietary curcumin supplementation significantly improved the dressing percentage and breast yield in heat-stressed broiler chickens. This may be attributed to the ability of dietary curcumin to decrease the level of protein oxidation in the breast muscles and minimize the loss of cellular fluids in stressed broiler chickens [35]. Consistent with these findings, Hang et al. [36] noticed a linear increase in the percentage of breast fillet with increasing levels of curcuminoids in broiler diets. Meanwhile, others suggested that the dressing percentage was not significantly affected by addition of 0.5% turmeric powder to broiler diets [37]. Excessive levels of abdominal fat is one of the major concerns in the broiler industry [38]. Herein, curcumin supplementation significantly reduced the percentage of abdominal fat in heat-stressed broilers, probably due to the ameliorative effect of curcumin on energy metabolism, and thus no need to store excess energy as fat deposits. Similarly, Nouzarian et al. [39] reported a significant decrease abdominal fat in broilers fed diets supplemented with turmeric powder (38.28 mg/kg curcumin content). Other researchers suggested that the different levels of curcuminoids in the broiler diets significantly reduced the percentage of abdominal fat [36]. Rajput et al. [22] did not report significant effects on the percentage of abdominal fat, when added up to 150 mg/kg pure curcumin in the broiler diets.

The primary concern of heat stress in broiler industry is the deleterious effect on muscle oxidative stability, duration of shelf life and meat composition that influence consumer acceptability [40]. In this context, dietary curcumin supplementation herein reduced the levels of MDA in the breast and thigh muscles, suggesting improved muscle oxidative stability in heat-stressed broilers. This may be attributed to the proven antioxidant properties of curcumin, which neutralizes the deleterious effects of free radicals associated with the oxidative heat stress [41]. In addition, other researchers suggested that curcumin enhances the transfer of antioxidant compounds involved in the reduction of MDA levels in the muscles [42]. Zhang et al. [43] demonstrated that curcumin supplementation at a rate of 100–200 mg kg^−1^ diet prevent the increase of MDA in heart tissues of heat-stressed broiler chickens.

Over the past few decades, the interest for meat products with beneficial effects on human health has been grown. Hence, broiler meat with its superior contents of unsaturated FAs is the best choice. In the present study, dietary curcumin supplementation reduced the contents of saturated FAs in the breast and thigh muscles of heat-stressed broiler chickens. Similar findings were reported by Daneshyar et al. [44], who stated that the addition of curcuma powder (0.75%) to broiler diets decreased the levels of saturated FAs in thigh muscles. Meanwhile, others suggested that curcuminoids supplementation did not alter the levels of saturated FAs in the breast and thigh muscles of Korat chickens [36]. Herein, the current study demonstrated the ability of dietary curcumin to restore the levels of MUFAs and PUFAs in the breast and thigh muscles of heat-stressed broiler chickens. This may be attributed to the antioxidant activities of curcumin, which prevent oxidation of labile unsaturated FAs. In this context, Gnoni et al. [45] confirm the ability of antioxidant-rich diets to modulate the activities of 9-desaturase enzyme complex, and consequently inhibit saturation process of FAs. Therefore, curcumin is likely to mediate in the regulation of the biosynthesis of PUFAs in broiler muscles. These findings agreed with that of Hang et al. [36], who suggested that broiler diets enriched with 20 mg kg^−1^ curcuminoids increased the levels of linoleic acid and total n-6 PUFAs in the breast muscles.

Heat stress is usually associated with accumulation of ROS in the mitochondria, with subsequent damages of lipids and DNA structures. As thermal stress is prolonged, the mitochondrial homeostasis is disturbed and ATP synthesis is decreased [12]. In the present study, the dietary curcumin supplementation improved the levels of ATP in liver tissues of heat-stressed broiler chickens. This may be attributed to the ability of curcumin to increase oxygen consumption and significantly decrease the levels of lipid and protein oxidation in liver mitochondria [46]. Together, the present study proved the ability of curcumin to scavenge the free radicals that could disturb the mitochondrial functions. Soto and Smith [47] demonstrated that the ATP level is positively correlated with the cellular mtDNA copy number. In this context, Zhang et al. [43] suggested that dietary curcumin could maintain the mtDNA copy number, and thus increased the ATP levels in liver tissues of heat-stressed broilers. Although previous studies demonstrated that acute heat stress reduced the level of Na^+^-K^+^-ATPase in the intestinal mucosa of chickens [48], the prolonged heat stress in the present study significantly increased the levels of Na^+^-K^+^-ATPase in the liver tissues of broilers, probably as a compensatory mechanism to alleviate the oxidative damages and restore the energy molecules. Coenzyme Q10; a natural lipophilic compound; regulates the process of oxidative phosphorylation in the mitochondria and acts as an antioxidant [49]. Furthermore, it is involved in the synthesis of ATP molecules and the regulation of bioenergetics pathway [50]. In the present study, supplemental curcumin improved the level of CoQ10 in liver tissues in heat-stressed broilers. This may be explained due to the ability of curcumin to improve the antioxidant properties in heat-stressed broiler chickens. In this context, Xu et al. [51] noticed that CoQ10 could reduce the oxidative damages of chicken myocardial cells. Additionally, treatment with CoQ10 could urge the expression of Hsp70 in heat-stressed broiler chickens [52].

Serotonin is a one of the main neurotransmitters controlling the neurological functions of the brain and nervous system. Moreover, it acts as an important messenger in the digestive tract and in the regulation of gastrointestinal motility and visceral sensation [33]. In this context, it is widely accepted that the level of brain serotonin is compromised when birds exposed to stressful conditions. In the current study, supplemental curcumin improved the level of serotonin in brain tissues of heat-stressed broilers. This may explain the increased FI of heat-stressed broilers when fed a curcumin-supplemented diet. Raybould [53] also suggested that serotonin plays a crucial role in controlling the contractility of smooth muscle in the digestive tract, as well as the activity of secretory epithelial cells. Consistent with our findings, Buraczewska et al. [54] stated that the supplementation of crystalline tryptophan to broiler diets improved the level of serotonin in brain tissues, and consequently increased the feed intake of broiler chickens. On the contrary, Denbow et al. [55] reported that tryptophan supplementation increased the level of brain serotonin in turkeys, but it had no effect on feed intake.

## 5. Conclusions

It could be concluded that dietary curcumin supplementation at a rate of 100 mg kg^−1^ may improve feed intake, carcass yield, and muscle oxidative stability, as well as the levels of unsaturated FAs (MUFAs and PUFAs) in the breast and thigh muscles of heat-stressed broiler chicken. Furthermore, supplemental curcumin could restore the levels of ATP and CoQ10 in liver tissues, as well as the brain serotonin, in heat-stressed broilers. The present outcomes may be helpful to adjust the appropriate strategies to reduce the adverse effects of chronic heat stress in broiler chickens.

## Figures and Tables

**Table 1:** Calculated chemical analysis of the corn-soybean basal diets of broiler chickens.

Calculated Analysis	Starter Period (1–21 Days)	Grower-Finisher Period (22–42 Days)
ME (KJ/kg)	12,342	12,949
Crude protein (%)	22.40	19.75
Calcium (%)	1.05	1.05
Available phosphorus (%)	0.45	0.45
Lysine (%)	1.18	1.14
Methionine (%)	0.48	0.45

ME: metabolizable energy.

**Table 2:** Effect of dietary curcumin supplementation on feed intake and carcass traits of heat-stressed broiler chickens.

Parameters (%)	Experimental Groups				
	^1^ T_1_	^2^ T_2_	^3^ T_3_	SEM ^4^	*p*-Value
^5^ ADFI (g)	117.5 ^a^	102.2 ^b^	116.7 ^a^	2.87	0.048
Dressing percentage	75.4 ^a^	68.5 ^b^	75.2 ^a^	1.13	0.010
Breast	42.1 ^a^	35.2 ^b^	40.9 ^a^	0.98	0.004
Legs	32.8	37.7	34.4	0.93	0.070
Liver	2.26	2.12	2.32	0.07	0.099
Heart	0.38	0.36	0.43	0.02	0.296
Abdominal fat	0.63 ^b^	0.95 ^a^	0.56 ^b^	0.05	0.017

^1^ thermoneutral group; ^2^ heat-stressed group; ^3^ curcumin-supplemented group; ^4^ standard error of means; ^5^ standard average daily feed intake. ^a,b^ Values within a row with different superscripts differ significantly.

**Table 3:** Effect of dietary curcumin supplementation on fatty acid profile (g/100 g) of breast muscles in heat-stressed broiler chickens.

Fatty Acids	Experimental Groups				
	^1^ T_1_	^2^ T_2_	^3^ T_3_	SEM ^4^	*p*-Value
Myristic (C_14:0_)	0.76 ^b^	0.97 ^a^	0.80 ^b^	0.04	0.001
Palmitic (C_16:0_)	30.8 ^b^	37.6 ^a^	33.2 ^b^	1.64	0.007
Stearic (C_18:0_)	12.28	12.98	12.31	0.59	0.159
Myristoleic acid (C_14:1_)	1.13 ^a^	0.83 ^b^	1.18 ^a^	0.04	0.009
Palmitoleic (C_16:1_)	1.22 ^a^	0.92 ^b^	1.15 ^a^	0.08	0.001
Oleic (C_18:1_)	20.7 ^b^	15.5 ^c^	23.4 ^a^	1.06	0.001
Linoleic (C_18:2n6_)	15.85 ^a^	11.31 ^b^	16.22 ^a^	0.74	0.016
*α*-linolenic acid (C_18:3n3_)	0.91	0.85	0.95	0.02	0.093
Docosahexaenoic acid (C_22:6n3_)	0.63 ^a^	0.40 ^b^	0.61 ^a^	0.02	0.001
Eicosapentaenoic acid (C_20:5n3_)	0.71 ^a^	0.49 ^b^	0.72 ^a^	0.03	0.035

^1^ thermoneutral group; ^2^ heat-stressed group; ^3^ curcumin-supplemented group; ^4^ standard error of means. ^a,b^ Values within a row with different superscripts differ significantly.

**Table 4:** Effect of dietary curcumin supplementation on fatty acid profile (g/100 g) of thigh muscles in heat-stressed broiler chickens.

Fatty Acids	Experimental Groups				
	^1^ T_1_	^2^ T_2_	^3^ T_3_	SEM ^4^	*p*-Value
Myristic (C_14:0_)	1.23	1.34	1.25	0.04	0.143
Palmitic (C_16:0_)	27.9 ^c^	31.7 ^a^	30.2 ^b^	1.77	0.001
Stearic (C_18:0_)	10.59 ^b^	11.85 ^a^	10.78 ^b^	0.38	0.048
Myristoleic acid (C_14:1_)	1.09 ^a^	0.83 ^b^	1.16 ^a^	0.04	0.001
Palmitoleic (C_16:1_)	1.98 ^a^	1.57 ^b^	2.08 ^a^	0.12	0.009
Oleic (C_18:1_)	28.9 ^a^	21.8 ^b^	28.4 ^a^	1.04	0.013
Linoleic (C_18:2n6_)	20.24 ^a^	13.09 ^c^	18.52 ^b^	0.45	0.006
*α*-linolenic acid (C_18:3n3_)	0.59	0.65	0.60	0.02	0.086
Docosahexaenoic acid (C_22:6n3_)	0.51 ^a^	0.36 ^c^	0.48 ^b^	0.02	0.001
Eicosapentaenoic acid (C_20:5n3_)	0.88 ^a^	0.57 ^c^	0.81 ^b^	0.04	0.026

^1^ thermoneutral group; ^2^ heat-stressed group; ^3^ curcumin-supplemented group; ^4^ standard error of means. ^a,b,c^ Values within a row with different superscripts differ significantly.

## Data Availability

All data generated or analyzed during this study are included in this published paper.
